# Presentation and Surgical Management of Duodenal Duplication in Adults

**DOI:** 10.1155/2015/659150

**Published:** 2015-12-30

**Authors:** Caroline C. Jadlowiec, Beata E. Lobel, Namita Akolkar, Michael D. Bourque, Thomas J. Devers, David W. McFadden

**Affiliations:** ^1^University of Connecticut General Surgery Residency Program, Farmington, CT 06030, USA; ^2^Connecticut Children's Medical Center, Department of Pediatric Surgery, Hartford, CT 06106, USA; ^3^University of Connecticut Health Center, Division of Gastroenterology, Farmington, CT 06030, USA; ^4^Department of Surgery, University of Connecticut Health Center, Farmington, CT 06030, USA

## Abstract

Duodenal duplications in adults are exceedingly rare and their diagnosis remains difficult as symptoms are largely nonspecific. Clinical presentations include pancreatitis, biliary obstruction, gastrointestinal bleeding from ectopic gastric mucosa, and malignancy. A case of duodenal duplication in a 59-year-old female is presented, and her treatment course is reviewed with description of combined surgical and endoscopic approach to repair, along with a review of historic and current recommendations for management. Traditionally, gastrointestinal duplications have been treated with surgical resection; however, for duodenal duplications, the anatomic proximity to the biliopancreatic ampulla makes surgical management challenging. Recently, advances in endoscopy have improved the clinical success of cystic intraluminal duodenal duplications. Despite these advances, surgical resection is still recommended for extraluminal tubular duplications although combined techniques may be necessary for long tubular duplications. For duodenal duplications, a combined approach of partial excision combined with mucosal stripping may offer advantage.

## 1. Introduction

Duodenal duplications in adults are exceedingly rare and their diagnosis remains difficult [1–3]. Treatment of duplications has traditionally involved surgical resection; however, for duodenal duplications, the anatomic proximity to the biliopancreatic ampulla makes surgical management challenging [4–6]. In our own experience, we recently encountered a symptomatic tubular duodenal duplication in an adult. Review of available literature finds that much of our knowledge of gastrointestinal duplications comes from pediatric case series [1, 7–12]. Here, we report our experience with an adult duodenal duplication and review embryology, clinical implications, and surgical management.

## 2. Case Discussion

**Figure 1:**
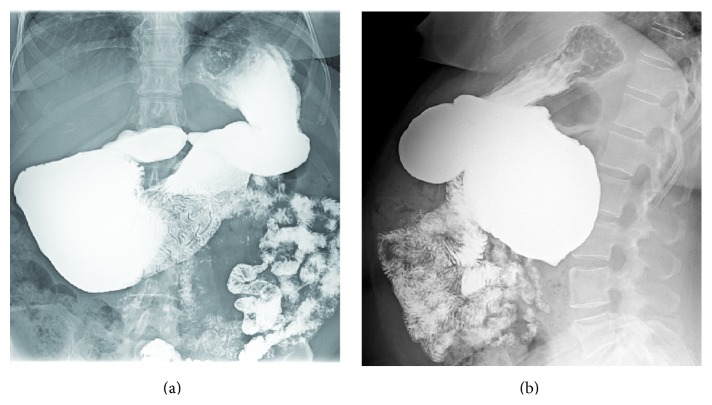
Representative images from the preoperative small bowel follow-through. There appeared to be marked abnormal dilation involving the duodenal C-loop. Of note, there was vigorous peristaltic activity involving this dilated loop although the peristaltic activity was disordered with a “to and from” movement of the barium and a marked delay in emptying into jejunum. The finding of peristaltic activity, although disordered, argued against the possibility of this being a duodenal diverticulum. (a) AP image. (b) Lateral image.

**Figure 2:**
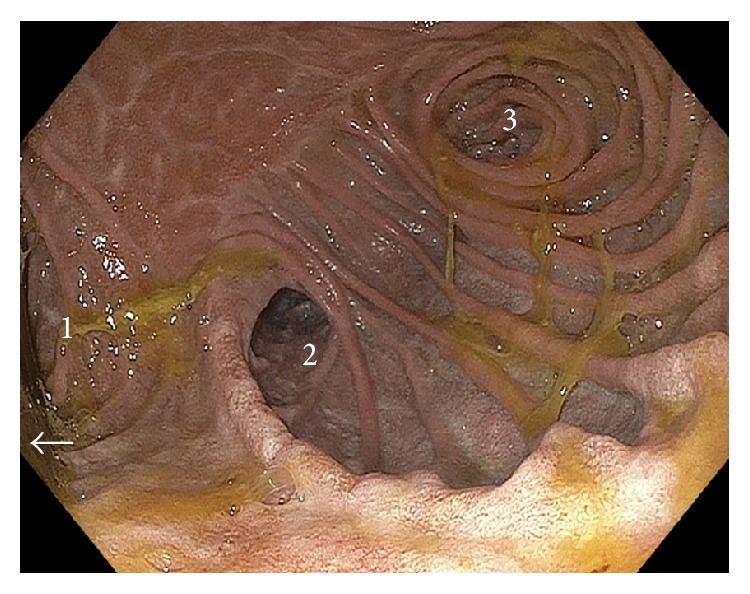
Preoperative endoscopic imaging. The second and third portions of the duodenum were noted to be markedly dilated with an accompanying finding of three downstream orifices. At this time, it was felt that the medial orifice (1) was the duplication and the lateral orifice (2) was the true lumen and that orifice (3) represented a distal common channel. White arrow denotes area of biliopancreatic ampulla which was proximal to the duplication.

A 59-year-old female was referred for evaluation secondary to several months of worsening postprandial abdominal pain, early satiety, reflux, and unplanned weight loss. The patient's laboratory values were unremarkable, and past medical and surgical history were noncontributory. Radiologic evaluation included a small bowel follow-through (Figure 1). Results of this study raised question of an abnormality involving the duodenal sweep. The duodenal C-loop was noted to be markedly dilated. At this time, two clinical diagnoses were considered. The first being that this duodenal dilation was occurring secondary to a stricture or an extrinsic compression in the fourth portion of the duodenum or at the duodenal-jejunal junction. The second possibility was that this finding represented some sort of enteric duplication. At this time, an upper endoscopy was performed (Figure 2). Findings from the endoscopy confirmed marked dilation to the duodenal C-loop. Bile pooling was noted to accompany this dilation. Surprisingly, three downstream orifices were found just distal to the biliopancreatic ampulla; these findings were again suggestive of a duodenal duplication. At that time, a surgical referral was obtained and the decision was made to proceed with operative exploration.

**Figure 3:**
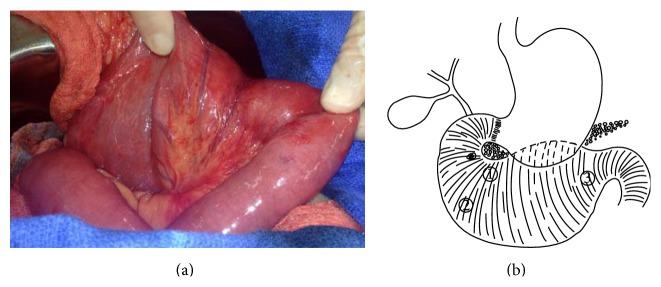
(a) Intraoperative photograph showing the duodenal duplication. (b) Schematic representation of intraoperative findings; included numbers correlate with endoscopic findings shown in Figure 2.

**Figure 4:**
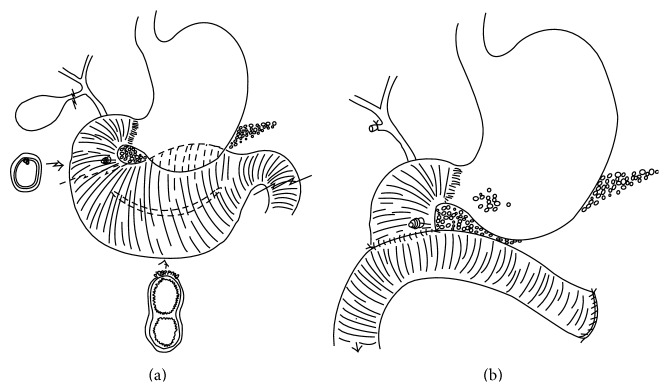
(a) Schematic of operative proceedings. The cystic duct was identified and a biliary Fogarty catheter was inserted into the duodenum. A duodenotomy was created and point of transection was chosen just distal to the ampulla so as to fully resect the duplication. The distal resection line occurring at the jejunum just beyond the ligament of Treitz. (b) Schematic of end-to-side duodenojejunostomy used for operative reconstruction.

The patient underwent an open abdominal exploration. Initial inspection found the second and third portions of the duodenum to be markedly dilated as had been observed on prior imaging (Figures 3(a)-3(b)) with a normal appearing distal jejunum. The retroperitoneal attachments of the duodenum were then taken down. The gallbladder was removed using a standard top-down approach; the cystic duct was identified and a biliary Fogarty balloon catheter was inserted into the duodenum so as to clearly delineate the location of the ampulla. Further medial mobilization of the duodenum was then performed, and at this time, it became apparent that the duplication extended superiorly, anterior to the body of the pancreas. Repeat intraoperative endoscopy was performed. Again, three orifices were confirmed to be just distal to the ampulla. A planned enterotomy was then created on the anterior surface of the duplication just distal to the ampulla; at this time it became apparent that one of these lumens was the true lumen, which connected to the distal jejunum. The other two orifices were lumens involving the duplication. Following clear delineation of anatomy, the tubular duodenal duplication was fully mobilized and resected (Figure 4(a)). A point of transection was chosen just distal to the ampulla with the distal resection line occurring at the jejunum. Accordingly, a hand-sewn end-to-side duodenojejunostomy was then fashioned (Figure 4(b)). In combining preoperative imaging and intraoperative findings, the final defined anatomy was consistent with that of a tubular duodenal duplication of the third and fourth portions of the duodenum.

## 3. Embryology

**Figure 5:**
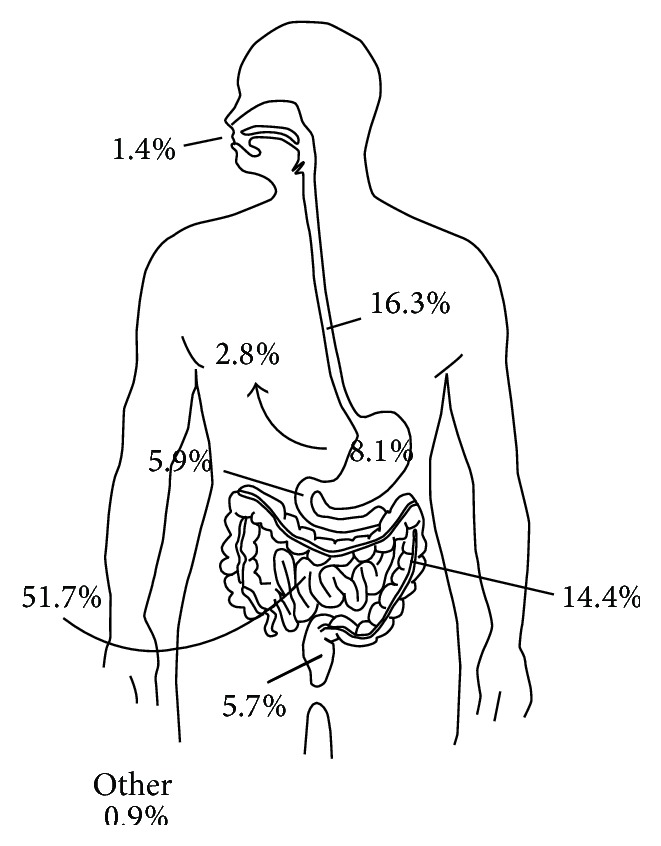
Alimentary tract duplications.

Duplications of the gastrointestinal (GI) tract are rare congenital anomalies that occur in either cystic or tubular form. Because of their relative infrequency, they tend to be clinically challenging with regard to diagnosis and treatment. Features common to all enteric duplications include their intimate attachment to the GI tract, epithelial mucosal lining, and a well-developed smooth muscle layer [7]. Duplications may or may not share a common communication with the native GI tract, and they may likewise be multiple. To date, the cause of GI duplications remains debated. The split notochord theory is commonly used to explain thoracic duplications where it is believed that there is incomplete separation of the notochord from the GI endoderm [7]. Alternatively, enteric duplications are hypothesized to arise as a result of recanalization errors involving the neonatal solid GI tract [2, 7, 8, 13]. Enteric duplications as a whole are believed to occur with an incidence of 1 per 4000–5000 live births [1, 3]. In comparison to other alimentary tract duplications, duodenal duplications are comparatively rare. In order of descending approximated frequency, jejunoileal duplications occur most commonly (52%), followed by esophageal (17%), colonic (14%), gastric (8%), duodenal (6%), and rectal (6%) duplications (Table 1 and Figure 5) [4, 9–12, 14–30].

## 4. Clinical Findings

Most enteric duplications are identified by the age of two years, with less than thirty percent being diagnosed in adults [3]. The large majority of duodenal duplications are cystic and intraluminal [31]. They most commonly arise from the mesenteric border of the second and third portions of the native duodenum, and accordingly, there is a tendency for them to be closely associated with both the pancreatic and biliary ducts. By comparison, extraluminal tubular duodenal duplications are rare [31, 32]. Cystic duodenal duplications are typically fluid-filled and however may, on occasion, contain gallstones, bile, or pancreatic fluid [32, 33]. Both computer tomography (CT) and ultrasound are useful imaging modalities [26, 28]. Ultrasound of cystic duplications should reveal an anechoic fluid-filled double-walled cyst composed of an inner hyperechoic rim of mucosa-submucosa and an outer hypoechoic layer of smooth muscle consistent with the muscularis propria; in contrast, although frequently difficult to capture, a classically identifying feature of tubular duplications is peristalsis [34]. For CT imaging, oral contrast used with CT imaging can be helpful as it will not fill a cystic duplication because of lack of communication with the gastrointestinal tract but rather may delineate it by demonstrating compression effect on adjacent structures whereas a tubular duplication would be expected to fill [29].

In adults, duodenal duplications remain difficult to diagnose, as presenting symptoms tend to be nonspecific [2]. With regard to duodenal duplications, commonly reported presenting symptoms include abdominal pain with weight loss, nausea, vomiting, and reflux. Accompanying clinical presentations include pancreatitis, biliary obstruction, gastrointestinal bleeding from ectopic gastric mucosa, and malignancy, and of these, pancreatitis appears to be the most frequent [32–38].

## 5. Treatment Options

Review of earlier literature highlights the complexity and surgical difficulty in treating enteric duplications. It is important to note that management of cystic intraluminal and tubular extraluminal duodenal duplications typically varies. Cystic intraluminal duplications are increasingly more amendable to drainage alone whereas extraluminal tubular duplications typically necessitate formal surgical resection.

Historically, prior attempts to create a drainage limb while leaving an intraluminal cystic duplication intact via a surgical cystjejunostomy resulted in incomplete long-term success primarily because of retained heterotrophic gastric tissue and long-term risk of peptic ulceration, bleeding, or malignancy [4, 5, 39]. Recent advances in endoscopy have expanded on this idea with successful drainage accomplished via endoscopic marsupialization [39]. Review of the literature finds that there are eight children who underwent endoscopic management and remained asymptomatic after mean follow-up of over seven years, thus suggesting that this may be a safe and effective technique [40, 41]. Concerns regarding malignancy or bleeding from peptic ulceration still exist in this setting, as endoscopic therapy does not always result in complete ablation of the cyst mucosa; this risk, however, appears to be low.

In contrast, current surgical understanding and practice still largely recommends resection for tubular extraluminal duplications. Complete resection for short segmental duplications may be possible whereas combined techniques may be necessary for longer segmental duplications. Duodenal duplications, in particular, pose a unique challenge as their anatomic proximity to the ampulla makes surgical management more complex. Of note, duodenal duplications typically arise just distal to the biliopancreatic ampulla in comparison to choledochoceles which are typically found proximal [41]. A combined approach of partial excision combined with mucosal stripping offers one such solution [5, 6, 22]. From the pediatric literature, mucosal stripping is a practical surgical option as it removes the secretory mucosa and also reduces the future risk of peptic ulceration and malignancy. Of note, during this process, the shared blood supply between the duplication and the native bowel must be protected and preserved so as to avoid the need for resection [3].

## 6. Conclusion

Despite a large historic experience, duodenal duplications in adults continue to be rare and their diagnosis remains difficult. Clinical symptoms in adults are nonspecific and potential risks, if untreated, including risk of peptic ulceration and malignant transformation. The treatment of duplications has traditionally involved surgical resection; however, for duodenal duplications, the anatomic proximity to the ampulla makes surgical management more challenging. For intraluminal cystic duodenal duplications, advances in endoscopy have changed current practice; however management of extraluminal tubular duplications remains challenging. In this setting, a combined approach of partial excision combined with mucosal stripping may offer advantage.

## Figures and Tables

**Table 1:** Alimentary tract duplications.

Ref.	Pub. yr.	Number of patients	Location								
			Oral	Esophageal	Thoracoabdominal	Gastric	Duodenal	Jejunal and ileal	Colonic	Rectal	Other
[7]	1935	90	—	10	—	6	8	59	3	3	1
[8]	1953	67	1	13	3	2	4	32	9	4	—
[9]	1956	25	—	5	—	4	2	16	5	—	—
[10]	1960	28	—	7	—	1	3	16	4	2	—
[11]	1961	38	1	6	2	1	—	18	6	4	—
[12]	1966	8	—	1	1	—	—	6	—	—	—
[13]	1970	23	—	4	2	1	—	9	7	—	—
[14]	1971	37	3	4	—	3	4	20	4	—	—
[1]	1978	64	—	15	1	6	6	34	12	2	2
[15]	1981	53	—	8	2	8	1	32	4	5	—
[16]	1988	11	—	1	—	1	2	4	2	1	—
[17]	1988	17	—	6	—	1	—	5	8	—	—
[18]	1989	96	1	20	3	8	2	47	15	5	—
[19]	1994	14	—	8	1	—	1	1	3	1	—
[20]	1995	72	2	15	6	10	3	21	10	6	4
[21]	1995	27	2	—	—	3	1	9	8	6	—
[22]	1996	17	—	2	—	1	—	14	3	—	—
[23]	2000	38	1	7	2	1	3	17	9	2	—
[24]	2000	12	—	—	—	3	1	8	—	1	—
[25]	2003	73	—	—	—	6	7	51	5	4	—

	**Total**	810	11 (1.4%)	132 (16.3%)	23 (2.8%)	66 (8.1%)	48 (5.9%)	419 (51.7%)	117 (14.4%)	46 (5.7%)	7 (0.9%)

Thoracoabdominal, intrathoracic duplication originating from below the diaphragm.
